# Sphingolipids and Epoxidized Lipid Metabolites in the Control of Gut Immunosurveillance and Allergy

**DOI:** 10.3389/fnut.2016.00003

**Published:** 2016-01-27

**Authors:** Jun Kunisawa, Hiroshi Kiyono

**Affiliations:** ^1^Laboratory of Vaccine Materials, National Institutes of Biomedical Innovation, Health and Nutrition (NIBIOHN), Osaka, Japan; ^2^Graduate School of Medicine, Osaka University, Suita, Japan; ^3^Graduate School of Pharmaceutical Sciences, Osaka University, Suita, Japan; ^4^Graduate School of Dentistry, Osaka University, Suita, Japan; ^5^Graduate School of Medicine, Kobe University, Kobe, Japan; ^6^Division of Mucosal Immunology, International Research and Development Center for Mucosal Vaccines, The Institute of Medical Science, The University of Tokyo, Tokyo, Japan; ^7^Department of Immunology, Graduate School of Medicine, Chiba University, Chiba, Japan

**Keywords:** lipid, vaccines, allergy and immunology, inflammation, dietary fats

## Abstract

The intestinal immune system ingeniously balances the distinct responses of elimination and tolerance of non-self-substances for the creation and maintenance of homeostatic environments. Accumulating evidence has recently shown that various lipids, including dietary one, are involved in the regulation of intestinal immunity and are associated with biophylaxis and immune disorders. Recent advances in the lipidomics allow the identification of novel pathways of lipid metabolism and lipid metabolites for the control of intestinal immunity. In this paper, we describe the effects and functions of lipids, especially sphingolipids and new lipid metabolites originated from dietary oil on the immunomodulation and on the development and pathogenesis of allergic diseases in the intestine.

## Introduction

A major physiological function of the intestine is the digestion and absorption of food. Simultaneously, *Vibrio cholera*, *Escherichia coli*, rotavirus, and other pathogenic microorganisms hitch a ride on this essential life-sustaining activity to invade the gut. Intestinal tissue is thus simultaneously exposed to foreign matter that is harmful (including pathogenic microorganisms) and to the other that is beneficial to the body (including dietary components and commensal bacteria) (1). The intestine functions to recognize pathogenic microorganisms that have invaded the body as undesired foreign matter and actively eliminate them, while dietary components and gut commensal bacteria are determined to be beneficial, and the intestine is regulated to permit their uptake or coexistence in the body. The breakdown of this homeostatic maintenance system is a cause of the development of intestinal immune disorders, notably infectious diseases, inflammatory bowel disease, and food allergies (2).

Previous studies have identified a range of intracorporeal (e.g., cytokines and chemokines) and extracorporeal (e.g., dietary materials and commensal bacteria) factors involved in the regulation of intestinal immunity (3–5), and attention has focused on lipids as important molecules in this process (6, 7). Technological progresses in the mass spectrometry-based lipidomics enable us to identify new pathway and metabolites participating in the immune regulation.

In this paper, we describe lipid-mediated intestinal immunomodulation, including the role of dietary lipids and new class of lipid metabolites, in the physiological and disease conditions.

## Production of Canonical Pro- and Anti-Inflammatory Lipid Mediators from Essential Fatty Acids

Among the long chain-fatty acids, ω3 and ω6 fatty acids are essential fatty acids that cannot be produced within the body and thus must be ingested from food. The main ω6 fatty acid found in dietary oils is linoleic acid (LA) (8). This is metabolized in the body to arachidonic acid, which in turn is further metabolized to lipid mediators, such as prostaglandin and leukotriene, by the action of the enzymes cyclooxygenase (COX), lipoxygenase (LOX), and cytochrome P450 (CYP) (8). It is well known that the LA-originated metabolites (e.g., prostaglandin E1, E2, and D2, lipoxin A4) possess both pro- and anti-inflammatory actions by affecting various immune cells (e.g., macrophages, neutrophils, and T cells) and non-immune cells (e.g., epithelial cells), which is mediated by different types of receptors [e.g., EP1, EP2, EP3, EP4, D-type prostanoid (DP), chemoattractant receptor homologous molecule expressed on Th2 cells (CRTH2), and ALX] [reviewed in Ref. (9, 10)].

The prominent ω3 fatty acid found in dietary oils is α-linolenic acid (ALA), which has long been known to have anti-inflammatory properties (11, 12). Because LA and ALA are both converted to their respective metabolic products by the same enzymes (8), the mechanism of action of ALA was formerly believed to involve the competitive inhibition of lipid metabolism originating with LA. However, recent advances in lipidomics technology have shown that in addition to competitive inhibition, ALA also actively participates in bioregulation by becoming a precursor of lipid mediators that exhibit an anti-inflammatory action (6, 11). ALA is metabolized in the body into eicosapentaenoic acid (EPA) and docosahexaenoic acid (DHA), after which they are metabolized by COX, LOX, and CYP and converted into the lipid mediators (e.g., prostaglandin E3, E and D series of resolvins, maresins, and protectins) to exert their anti-inflammatory actions by inhibiting neutrophil infiltration, promoting phagocytosis of neutrophils by macrophages, and inhibiting IFN-γ, tumor necrosis factor (TNF)-α, and inducible nitric oxide synthase production (6, 11). The mechanisms underlying these actions include signal transduction *via* receptors (e.g., GPR40 and GPR120), gene expression (e.g., PPAR family and NFκB), and membrane characteristics (e.g., fluidity and lipid raft formation) (13, 14).

## Influence of Fatty Acids in Dietary Oils on the Development of Gut Immune Diseases

Epidemiological studies indicate the association of incidence of inflammatory disorders and infectious diseases with dietary fatty acid composition (15–17) and lipidomics analyses identified key lipid metabolites responsible for these responses (18, 19).

**Figure 1:**
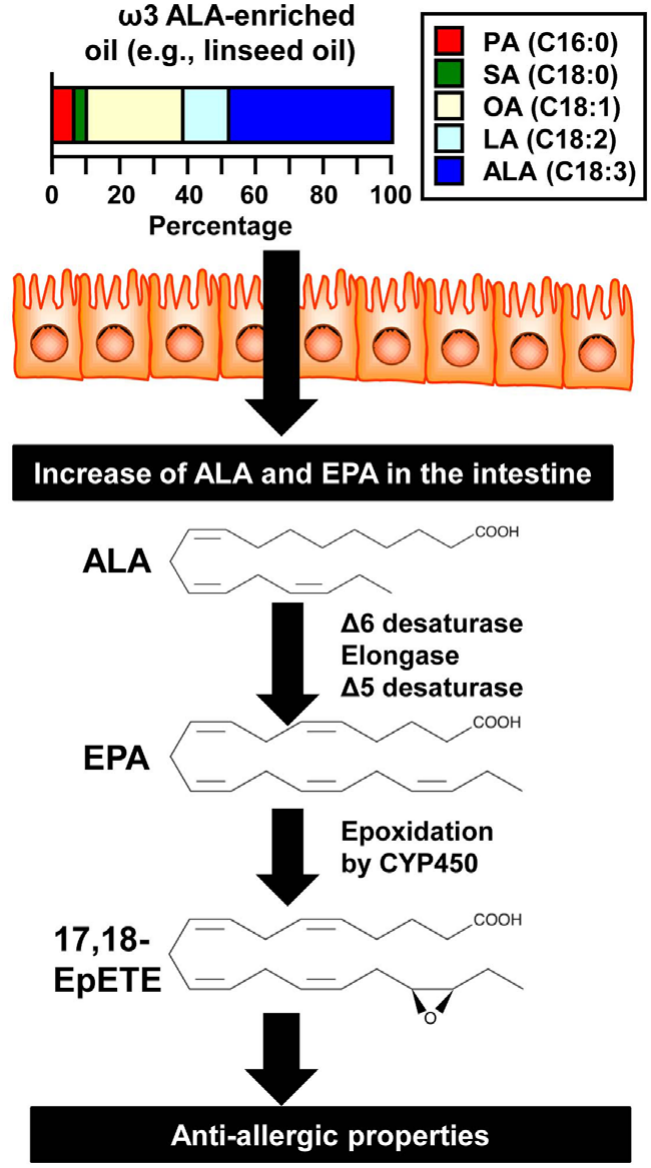
**Metabolic pathway in the generation of anti-allergic/inflammatory lipid mediators from the dietary oil**. ALA is enriched in some kinds of dietary oil (e.g., linseed oil) and absorbed into the intestinal tissues where it is metabolized into EPA after several reactions mediated by various enzymes (e.g., elongase and desaturases). EPA is epoxidized by CYP450 to generate 17,18-EpETE, which exerts anti-allergic properties in the intestine. Abbreviations: ALA, α-linolenic acid; CYP, cytochrome P450; EPA, eicosapentaenoic acid; EpETE, epoxy eicosatetraenoic acid; LA, linoleic acid; OA, oleic acid; PA, palmitic acid; SA, stearic acid.

In addition to inflammatory bowel diseases, food allergies are intestinal immune disorders that have become increasingly common in recent years. The food allergy occurs as type I allergic reactions, with the production of allergen-specific IgE as well as activation and degranulation of mast cells (20, 21). To examine the effect of dietary oil on the development of food allergy, we conducted animal experiments, using special chow that contained various dietary oils with a range of different fatty acid compositions, and found that differences in the fatty acid composition of dietary oils affected the development of food allergy (22). Different types of dietary oils vary greatly in their fatty acid compositions, and the soybean oil used in regular mouse chow contains approximately 50% LA and roughly 5% ALA. Linseed oil (also known as flaxseed oil), on the other hand, is known to have a high ALA content of approximately 60%, more than 10 times higher than that of soybean oil. We therefore tested the effect of differences in the proportions of ω6 (LA) and ω3 (ALA) fatty acids on the development of food allergy. Mice were fed with chow containing either 4% soybean oil (the oil used in normal chow) or linseed oil for 2 months and subjected to a food allergy model with ovalbumin (OVA) as the allergen. Mice maintained with linseed-oil-containing chow exhibited less diarrhea incidence caused by OVA-induced food allergy than did those raised on chow containing soybean oil (22). Similarly, dietary fish oil has the same effect on the intestinal allergy (23, 24). We further revealed that the fatty acid composition of intestinal tissue from these mice was correlated with the fatty acid compositions of their diets: the colons of mice raised on chow containing linseed oil contained higher concentrations of ALA acid and its metabolites EPA and DHA (Figure 1), whereas those of mice raised on chow containing soybean oil contained high levels of LA and its metabolite arachidonic acid (22). These results indicated that the relative proportions of ω3 and ω6 fatty acids in dietary oils determine the composition of ω3 and ω6 fatty acids and their metabolites in the intestines.

## Conversion of EPA to Anti-Allergic Lipid Metabolite for the Control of Food Allergy

As has already been mentioned, the physiological activity exhibited by the metabolites of EPA and DHA is now coming under the spotlight in the area of nutritional or food immunology (6, 11). We employed lipidomics technology to carry out a comprehensive analysis of fatty acid metabolites, allowing us to identify the fatty acid metabolites increased in the colons of mice raised on linseed-oil-containing chow. We found a marked increase in the content of 17,18-epoxyeicosatetraenoic acid (17,18-EpETE), a metabolite produced by the action of CYP on EPA as the substrate (Figure 1) (22). We then used synthetic 17,18-EpETE to test the anti-allergic effect of 17,18-EpETE and found that mice that received 17,18-EpETE administration exhibited the same reduced incidence of allergic diarrhea seen in mice fed linseed-oil-containing chow (Figure 1) (22).

17,18-EpETE is metabolized to 17,18-dihydroxyeicosatetraenoic acid (17,18-diHETE) by the action of epoxide hydrolase, which cleaves the epoxy ring (25). The colons of mice raised on linseed-oil-containing chow also contained high levels of 17,18-diHETE in addition to 17,18-EpETE. However, unlike 17,18-EpETE, 17,18-diHETE had almost no effect in suppressing allergic diarrhea (22), suggesting that 17,18-EpETE may be the active molecule in the EPA-derived lipid mediator that suppresses intestinal allergy. In light of previous debates about the possibility that epoxide hydrolase inhibition may improve circulatory disorders, such as hypertension and arteriosclerosis, as well as chronic inflammatory disorders, such as colitis and arthritis (26, 27), inhibiting the metabolic conversion of 17,18-EpETE to 17,18-diHETE by blocking the action of epoxide hydrolase might further suppress the development of intestinal allergy. CYP, which is a responsible enzyme to generate 17,18-EpETE from EPA, exists in a range of different subfamilies and types (28). Collectively, these findings suggest that it will be important not only to identify anti-allergic/anti-inflammatory lipids but also to consider the various metabolic pathways involved in lipid production, metabolism, and decomposition for the development of novel prevention and treatment strategies for allergies and inflammatory disorders based on the knowledge of lipid mediators and its related metabolites.

## Sphingosine 1-Phosphate as an Intestinal Immunomodulatory Factor for IgA Responses

**Figure 2:**
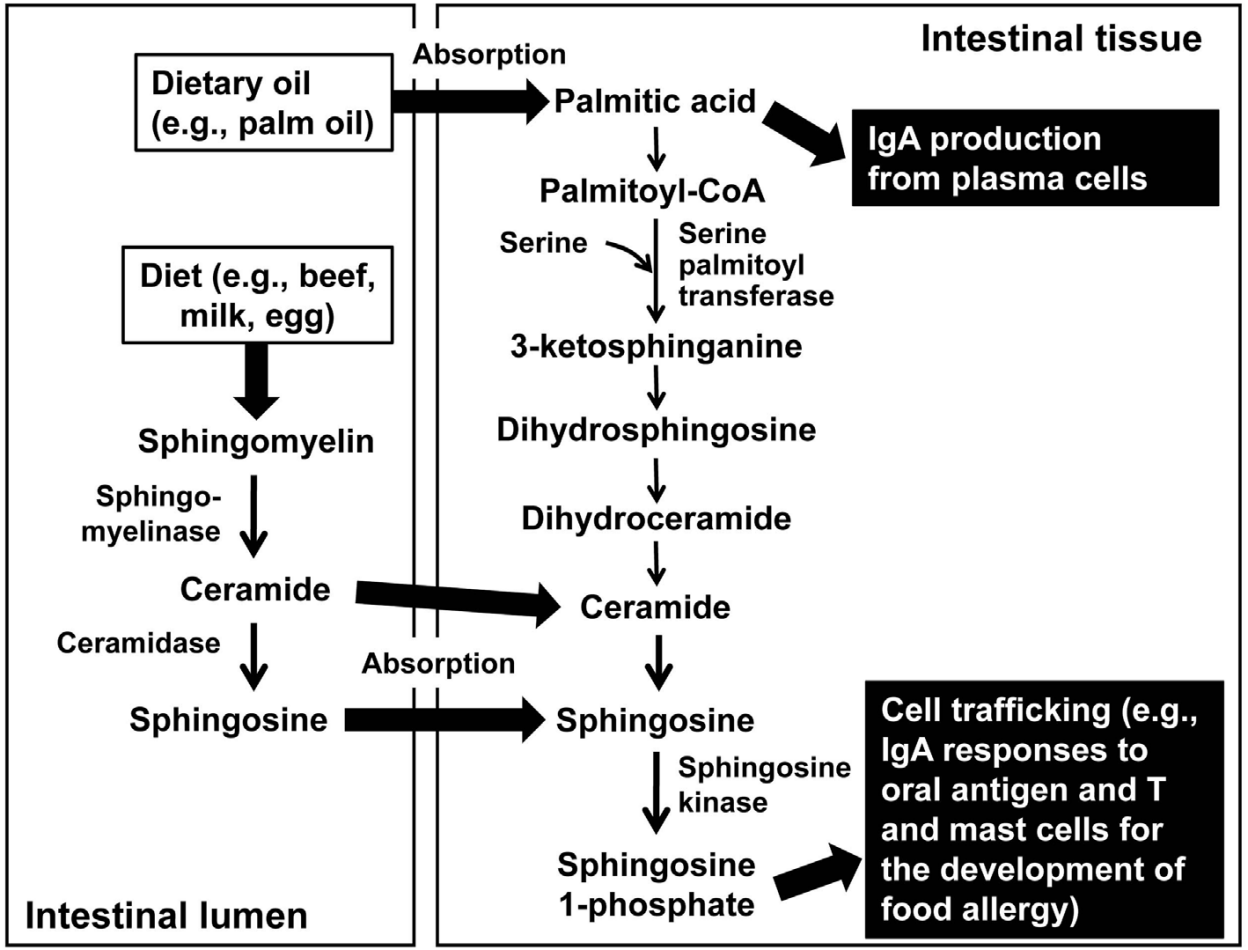
**Two pathways for the generation of sphingolipids in the control of immune responses in the intestine**. Palmitic acid is absorbed from dietary oil (e.g., palm oil) into the intestinal tissue where it directly stimulates IgA-producing plasma cells to enhance IgA production. Simultaneously, palmitic acid is metabolized into the sphingolipids, such as ceramide, sphingosine, and sphingosine 1-phosphate. In the other pathway, sphingomyelin abundantly present in the diets, such as beef, milk, and egg is metabolized into ceramide and then to sphingosine in the intestinal lumen. Both ceramide and sphingosine are absorbed into the intestinal tissue where they are further metabolized into the sphingosine 1-phosphate. Sphingosine 1-phosphate regulates cell trafficking and thus controls the IgA antibody responses to oral antigen. It also participates in the development of food allergy by controlling T and mast cell trafficking and/or growth.

Sphingosine 1-phosphate (S1P) is a type of sphingolipid that is a focus of attention as a lipid mediator involved in immunomodulation (29, 30). In the body, S1P is mainly generated *via* the sphingomyelin → ceramide → sphingosine → S1P pathway by the action of sphingomyelinase, ceramidase, and sphingosine kinase, respectively (Figure 2). Furthermore, S1P is returned to sphingosine by the action of phosphatase or is irreversibly decomposed by S1P lyase. The balance between production, decomposition, and metabolism is carefully regulated to form a gradient of S1P concentration in the body, which controls the cell trafficking, such as the emigration of thymocytes and lymphocytes from the thymus and secondary lymphatic tissues, respectively (31). Given that S1P is also involved in the immune reactions of a wide range of cells, including T cells, macrophages, dendritic cells, and vascular endothelial cells, it is attracting attention as an important lipid mediator responsible for key immune functions in the immunosurveillance and immune diseases (31).

We have previously shown that S1P plays important roles in physiological and pathological circumstances of the intestinal immune system (32). For example, S1P receptor expression is changed during B cell differentiation in the Peyer’s patches, a major organized mucosa-associated lymphoid tissue for the initiation of antigen-specific IgA immune responses (33). Upon the class switching to IgA^+^ B cells, naïve B cells decrease the S1P receptor expression, which allows their retention in the Peyer’s patches. Then, IgA^+^ B cells show the recovered expression of S1P receptors for the emigration from the Peyer’s patches. Similarly, peritoneal B cells, which contain B1 B and B2 B cells for the antibody production against T-independent and -dependent antigens, respectively, express high levels of S1P receptors and thus use the S1P for their trafficking into the intestinal compartments (34, 35). For this reason, mice administered FTY720 to inhibit the S1P pathway by inducing the internalization of S1P receptors exhibit a reduced intestinal IgA antibody reaction to oral vaccines (Figure 2) (33, 35).

Another example is trafficking of specialized T cells in the epithelial layers of the intestine, which are known as intraepithelial lymphocytes (IELs). They eliminate pathologic epithelial cells and maintain the function of “healthy” epithelial functions as the front line of defense (36). IELs include subtypes [e.g., T cells expressing T cell receptor (TCR) αβ or γδ] and display different S1P dependencies in the migration from the thymus to the intestinal epithelium. Indeed, FTY720 treatment resulted in the selective reduction of IELs expressing TCRαβ in the colon, whereas colonic IELs expressing TCRγδ were insensitive to FTY720 treatment, which provides immunological diversity at the front of body surface (37).

## Sphingosine 1-Phosphate Mediates the Development of Intestinal Immune Diseases

S1P has also been shown to contribute to the development of immune disorders. In an analysis of murine food allergy model that we mentioned above, we discovered that S1P involved in the occurrence of allergic diarrhea. Hence, the development of allergic diarrhea was prevented by FTY720 administration (38). In FTY720-treated mice, no change was noted in the allergen-specific IgE production, whereas infiltration of IL-4-producing pathogenic T cells into the colon was inhibited. Also, the increase of mast cells in the colon was also prevented in the mice receiving FTY720 treatment. In addition to the trafficking of mast cells, S1P is produced by mast cells, which regulate their maturation and phenotype, and responsiveness (39–41).

Similarly, FTY720 administration has also been shown to be effective in the prevention of intestinal inflammation by blocking S1P-dependent pathogenic cell trafficking (e.g., IFN-γ-producing T cells) (42). Thus, S1P plays a critical role in the control of trafficking of pathogenic cells, which could be a prospective target for the control of intestinal immune diseases (e.g., food allergy and intestinal inflammation).

## Sphingolipid Metabolism for the Induction of Immune Responses in the Intestine

In terms of sources of S1P and related sphingolipids in the intestine, the S1P precursor sphingomyelin is found in large quantities in foods, such as meat, milk, and eggs (Figure 2). Because almost all bacteria lack sphingolipids, there is almost no change in the sphingolipid content of the gut of germ-free animals when compared with conventional/specific pathogen-free mice (43). As such, most sphingolipids in the intestinal environment are likely to be derived from dietary sources. Dietary sphingomyelin is actually metabolized to ceramide and sphingosine by the action of a group of enzymes expressed in intestinal epithelial cells and absorbed, after which it is converted to S1P by sphingosine kinase (Figure 2) (44, 45). Consequently, high levels of sphingolipids are detected in the intestines (46).

Sphingolipids are also synthesized in the body *de novo via* a pathway that originates with palmitic acid (Figure 2). We have demonstrated that palmitic acid acts to increase antibody production. Mice raised on a special chow with an increased palmitic acid content or chow manufactured from palm oil, which has a high palmitic acid content, excreted more IgA antibodies in their feces (Figure 2) (47). These mice also exhibited an enhanced intestinal antigen-specific IgA antibody response to oral vaccines. We identified a pathway for this effect, in which palmitic acid acts directly on IgA antibody-producing cells and increases the generation of IgA antibodies (Figure 2) (47). Furthermore, we also identified a pathway in which the sphingolipids, generated when palmitic acid is metabolized by serine palmitoyltransferase, increase IgA antibody-producing cells in the colon (47). Taken together, the production, metabolism, and recognition of sphingolipids, mediated by intracorporeal and extracorporeal factors, are implicated in a wide range of intestinal immune reactions.

## Conclusion

Although the number of patients with food allergies, inflammatory bowel disease, and other mucosal immune disorders is increasing recently, little therapy and prevention are available now. Recent developments in lipidomics technology have made it possible to carry out comprehensive analyses of the lipid metabolism network in the body. Owing to these newly developed techniques, novel preventive and therapeutic target candidates for immune disorders have been identified. In addition, these technologies can be used for the development of lipid-based safe and effective adjuvant to enhance the vaccine effects. Thus, the formation of the lipid network, including dietary lipids, will become an increasingly important topic of research in the basic biology area as well as the drug and vaccine development for the control of immune disorders and infectious diseases.

## Author Contributions

JK and HK wrote the manuscript.

## Conflict of Interest Statement

The authors declare that the research was conducted in the absence of any commercial or financial relationships that could be construed as a potential conflict of interest.
